# Large, Chronic, Perineal Low-Grade Intraepithelial Lesion in an Immunocompetent Patient: A Case Report

**DOI:** 10.7759/cureus.109201

**Published:** 2026-05-19

**Authors:** Trevor Williams, Shashawna S Drum Christie, Navya N Malik, Bom K Bong, Azka T Iqbal, Frederick Tiesenga

**Affiliations:** 1 Medicine, St. George’s University, School of Medicine, St. George's, GRD; 2 Medicine, St. George's University, School of Medicine, St. George's, GRD; 3 Medicine, Caribbean Medical University, School of Medicine, Willemstad, CUW; 4 Internal Medicine, Community First Medical Center, Chicago, USA; 5 General Surgery, West Suburban Medical Center, Chicago, USA

**Keywords:** anogenital warts, condyloma acuminatum, human papillomavirus, low-grade squamous intraepithelial lesion, perineal lesion, secondary intention healing, surgical excision, wound healing

## Abstract

Condyloma acuminatum is a benign anogenital lesion that can vary in presentation from incidental findings to large, clinically significant masses. We present a case of a 53-year-old male with a medical history notable for obesity (BMI = 36.7 kg/m²) and a 20-pack-year smoking history. Additionally, the patient had a six-month history of a progressively enlarging perineal lesion identified incidentally during a routine screening colonoscopy. Examination revealed a dominant exophytic perineal mass measuring approximately 6 × 8 cm, along with three additional satellite lesions measuring approximately 2 × 2 cm each. The patient underwent surgical excision of all lesions, which were left to heal by secondary intention. Histopathologic evaluation demonstrated a low-grade squamous intraepithelial lesion consistent with condyloma acuminatum, with negative margins and no evidence of high-grade dysplasia or malignancy. Postoperative management included local wound care, nutritional optimization with high-protein intake and vitamin supplementation, and close clinical follow-up. At the one-week evaluation, the wounds demonstrated appropriate healing without evidence of infection. This case highlights the importance of thorough perineal examination, histopathologic confirmation of large lesions, and optimization of modifiable risk factors to support postoperative recovery.

## Introduction

Condyloma acuminatum is a common manifestation of infection with low-risk human papillomavirus (HPV), most frequently involving HPV subtypes 6 and 11 [1]. These lesions are classified histologically as low-grade squamous intraepithelial lesions (LSIL) under the Lower Anogenital Squamous Terminology (LAST) system and represent productive viral infection without high-grade dysplasia or invasive malignancy [2]. Anogenital warts affect approximately 1% of sexually active adults and remain among the most common clinical manifestations of HPV infection [1].

Although condyloma acuminatum is typically associated with sexual transmission, HPV infection may remain clinically latent for prolonged periods before lesion development. As a result, patients may present without identifiable behavioral risk factors despite harboring HPV-related disease [1].

While most lesions are small and amenable to topical therapy, large or multiple lesions require careful evaluation to exclude high-grade dysplasia or malignancy and may necessitate surgical excision for definitive treatment and histopathologic confirmation. This report describes a case of extensive perineal LSIL incidentally identified during colonoscopy positioning, highlighting surgical management and postoperative considerations in the context of modifiable host risk factors.

## Case presentation

A 53-year-old male presented for a routine screening colonoscopy with no prior colorectal cancer screening. During procedural positioning and perineal inspection, multiple lesions were identified. The patient reported first noticing a small lesion approximately six months earlier that had progressively enlarged. He denied pain, bleeding, drainage, pruritus, or systemic symptoms, including fever, weight loss, or malaise. He also denied high-risk sexual behaviors or prior sexually transmitted infections.

His past medical history was significant for hypertension, hyperlipidemia, obstructive sleep apnea, and chronic sciatica associated with degenerative disc disease. His body mass index (BMI) was 36.7 kg/m². He reported a 20-pack-year smoking history, occasional alcohol use, and intermittent marijuana use. The patient had no history of diabetes mellitus, sexually transmitted infections, malignancy, human immunodeficiency virus infection, or immunosuppressive medication use to suggest an underlying immunocompromised state.

Initial physical examination suggested a single dominant perineal mass; however, examination under anesthesia (during the colonoscopy) demonstrated multiple interconnected condylomatous lesions and smaller satellite masses surrounding the primary lesion. Evaluation of the lesions revealed a dominant verrucous, papillomatous perineal mass measuring approximately 6 × 8 cm, along with three additional satellite lesions measuring approximately 2 × 2 cm each. The lesions were non-tender, non-erythematous, and without drainage. No inguinal lymphadenopathy was appreciated.

**Figure 1:**
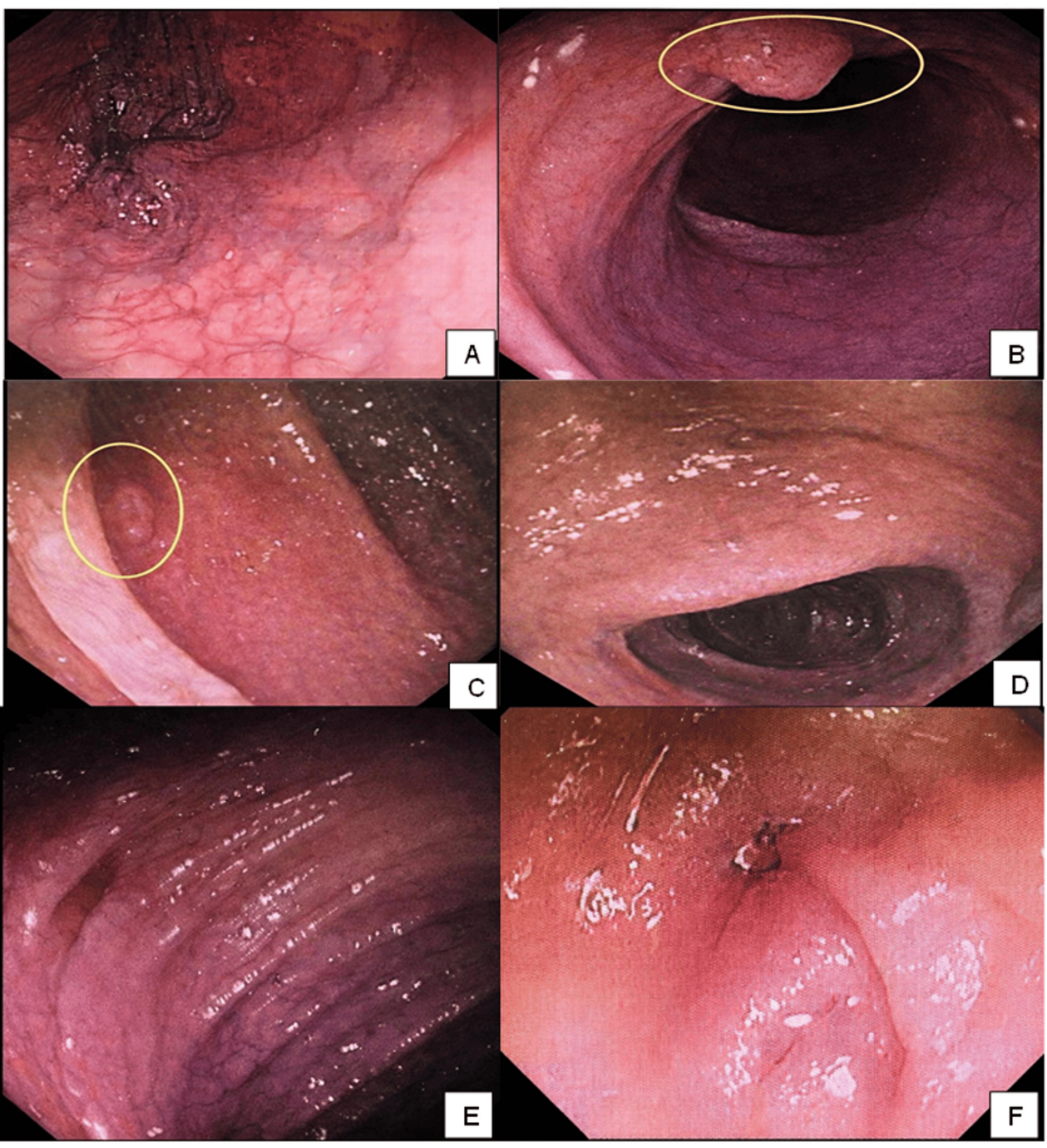
Colonoscopy images showing different pathological findings. A: Rectum showing internal hemorrhoids, characterized by engorged, bulging submucosal vascular cushions with preserved overlying mucosa and no active bleeding. B: Rectosigmoid junction demonstrating a sessile polypoid lesion (circled), consistent with one of multiple polyps identified in this segment; the surrounding mucosa appears intact without ulceration. C: Ascending colon with a discrete polyp (circled), appearing as a small, raised lesion with smooth contour, consistent with additional synchronous polyps noted during the examination. D: Transverse colon demonstrating a diverticulum, visualized as a sac-like mucosal outpouching with a well-defined opening and no surrounding inflammation. E: Descending colon showing another diverticulum, similar in appearance, without evidence of diverticulitis such as erythema, edema, or purulent material. F: Sigmoid colon demonstrates a diverticulum, identified by a small orifice within the colonic wall, again without signs of acute inflammation or complication.

During the colonoscopy procedure, multiple pathological features were identified, including rectal hemorrhoids and multiple polyps within the rectosigmoid junction and ascending colon. Additionally, a diverticulum was found within the transverse colon, descending colon, and sigmoid colon. Colonoscopy images are presented in Figure 1.

The patient was taken to the operating room for excision under general anesthesia. The dominant lesion was circumferentially excised through the skin and subcutaneous tissue. Three additional lesions were similarly excised. Hemostasis was achieved with electrocautery. Given the lesion size and anatomical location, the wounds were left open to heal by secondary intention to minimize tension and reduce the risk of dehiscence. All specimens were sent for histopathologic evaluation.

**Figure 2:**
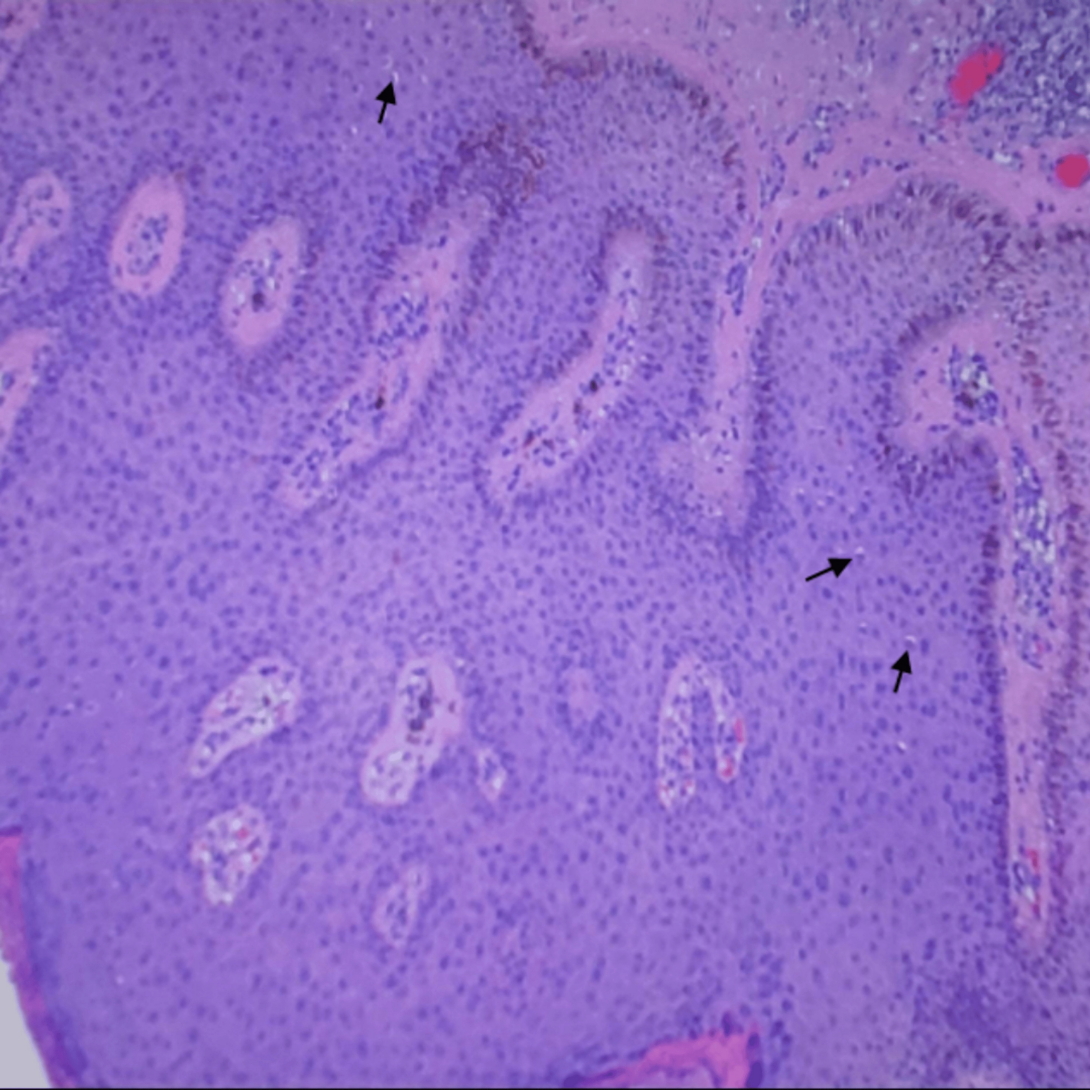
Histopathologic features of low-grade squamous intraepithelial lesion (LSIL). Hematoxylin and eosin (H&E)-stained section demonstrating acanthosis, papillomatosis, and koilocytic change consistent with condyloma acuminatum. Arrows indicate areas of koilocytosis characterized by perinuclear clearing and nuclear enlargement with irregular contours. No high-grade dysplasia or stromal invasion is present.

Histopathologic evaluation demonstrated papillomatosis, acanthosis, and koilocytic changes consistent with HPV-associated LSIL-condyloma acuminatum (Figure 2). No high-grade dysplasia or invasive carcinoma was identified, and resection margins were negative.

The patient tolerated the procedure well and was discharged with instructions for daily cleansing, sitz baths, and twice-daily wet-to-dry dressing changes to promote granulation tissue formation [3,4]. An antimicrobial dressing was utilized to maintain a moist wound environment and reduce bacterial colonization.

Nutritional optimization was emphasized, including multivitamin and vitamin C supplementation and a high-protein diet to support collagen synthesis and wound healing [3]. The patient was advised to avoid local trauma and abstain from sexual activity until healing was complete. At one-week follow-up, the wounds demonstrated appropriate healing without signs of infection. The patient reported minimal pain and no functional limitation. Follow-up was scheduled at two weeks and three months for continued monitoring.

## Discussion

Condyloma acuminatum is a benign epithelial proliferation caused primarily by low-risk HPV subtypes 6 and 11 and is classified histologically as LSIL under the LAST system [1,2]. Although these lesions have minimal malignant potential, recurrence remains common due to persistent viral reservoirs in surrounding epithelial tissue [1].

This case is notable for the incidental identification of multiple large perineal lesions during colonoscopy positioning rather than during evaluation for dermatologic or sexually transmitted disease. While sexual transmission is the primary route of HPV acquisition, viral latency is well established, and lesions may develop long after initial exposure [1].

The size and exophytic appearance of the dominant lesion raised consideration for Buschke-Löwenstein tumor (BLT), a rare, locally aggressive variant of condyloma acuminatum characterized by slow, expansile growth, local tissue destruction, and a tendency for recurrence [5]. However, histopathology demonstrating LSIL without features of a verrucous, pushing-border architecture is not consistent with BLT, which typically requires characteristic broad-based exophytic condylomatous proliferation with marked epithelial hyperplasia. In this case, the absence of these defining histopathologic features effectively excluded BLT as a diagnosis.

Host-related factors likely contributed to both lesion persistence and postoperative healing. Obesity is associated with impaired wound healing due to decreased vascularity and increased inflammatory response [6]. Additionally, smoking significantly impairs wound healing through vasoconstriction, reduced tissue oxygenation, and decreased fibroblast activity [7,8]. Smoking has also been associated with increased HPV persistence and recurrence [7].

Chronic wound formation and delayed healing likely contributed to the atypical presentation and persistence of this lesion. The patient’s obesity and tobacco use were clinically significant not only because of their known negative effects on tissue perfusion and wound healing, but also because both factors have been associated with impaired HPV clearance and persistent epithelial dysplasia [9-11]. Smoking is believed to promote HPV persistence through local immune suppression and oxidative tissue injury, while obesity has been linked to chronic inflammatory and immunomodulatory states that may impair antiviral immune responses [10-12]. In this patient, repeated local irritation, chronic inflammation, and impaired healing may have created a favorable microenvironment for persistent HPV-related dysplastic change and lesion progression. These host-related factors, therefore, likely contributed to both the chronicity of the wound and the persistence of the LSIL-associated perineal lesion.

The anatomic location of the lesion also likely contributed to its persistence and chronicity. The perineum, being intertriginous in nature, is particularly susceptible to chronic moisture exposure leading to maceration, frictional injury, bacterial contamination, and impaired aeration, all of which may promote epithelial breakdown and persistent HPV infection [13,14]. In the setting of obesity and smoking, these regional factors may further impair tissue healing and create a local environment that promotes prolonged inflammation, lesion progression, and lesion persistence [10-14].

Management options for condyloma acuminatum include topical and procedural therapies. Topical treatments such as imiquimod and podophyllotoxin are appropriate for smaller lesions but require prolonged therapy and demonstrate variable efficacy. In contrast, surgical excision provides immediate removal of bulky disease and allows histopathologic evaluation to exclude malignancy [1]. For large or multiple lesions, excision remains the preferred treatment.

Recurrence occurs in approximately 20-30% of cases, often within the first few months following treatment, emphasizing the importance of patient counseling and ongoing surveillance [1].

HPV vaccination represents an important preventive strategy. The nonavalent vaccine provides protection against HPV subtypes 6 and 11 and is recommended through shared decision-making for adults aged 27-45 years who may benefit from protection against previously unencountered HPV strains [1].

This case underscores the importance of thorough perineal examination during procedural positioning, particularly in patients with obesity, and highlights the role of surgical excision in managing large condylomatous lesions while addressing modifiable risk factors.

To our knowledge, incidental identification of extensive perineal LSIL during colonoscopy positioning in an asymptomatic patient without reported high-risk behaviors has rarely been described, emphasizing the importance of routine perineal inspection in procedural settings.

## Conclusions

This case highlights an extensive presentation of perineal LSIL in a patient without typical risk factors. Large condylomatous lesions may remain asymptomatic and undetected for extended periods. Surgical excision provides definitive management and diagnostic confirmation, while optimization of modifiable risk factors such as smoking and obesity is essential to improve healing and reduce recurrence risk. Continued surveillance and patient counseling remain critical for long-term management.

## References

[1] Workowski KA, Bachmann LH, Chan PA (2021). Sexually transmitted infections treatment guidelines, 2021. MMWR Recomm Rep.

[2] Darragh TM, Colgan TJ, Cox JT (2012). The Lower Anogenital Squamous Terminology Standardization Project for HPV-associated lesions: background and consensus recommendations from the College of American Pathologists and the American Society for Colposcopy and Cervical Pathology. Arch Pathol Lab Med.

[3] Hess CT (2019). Comprehensive patient and wound assessments. Adv Skin Wound Care.

[4] Bonham PA, Brunette G, Crestodina L (2022). 2021 guideline for management of patients with lower-extremity wounds due to diabetes mellitus and/or neuropathic disease: an executive summary. J Wound Ostomy Continence Nurs.

[5] Chu QD, Vezeridis MP, Libbey NP, Wanebo HJ (1994). Giant condyloma acuminatum (Buschke-Lowenstein tumor) of the anorectal and perianal regions. Analysis of 42 cases. Dis Colon Rectum.

[6] Pierpont YN, Dinh TP, Salas RE, Johnson EL, Wright TG, Robson MC, Payne WG (2014). Obesity and surgical wound healing: a current review. ISRN Obes.

[7] Moscicki AB, Hills N, Shiboski S (2001). Risks for incident human papillomavirus infection and low-grade squamous intraepithelial lesion development in young females. JAMA.

[8] Sørensen LT (2012). Wound healing and infection in surgery. The clinical impact of smoking and smoking cessation: a systematic review and meta-analysis. Arch Surg.

[9] Schabath MB, Villa LL, Lazcano-Ponce E, Salmerón J, Quiterio M, Giuliano AR (2012). Smoking and human papillomavirus (HPV) infection in the HPV in Men (HIM) study. Cancer Epidemiol Biomarkers Prev.

[10] Moscicki AB, Schiffman M, Burchell A (2012). Updating the natural history of human papillomavirus and anogenital cancers. Vaccine.

[11] Lee EY, Muller WJ (2010). Oncogenes and tumor suppressor genes. Cold Spring Harb Perspect Biol.

[12] Iyengar NM, Gucalp A, Dannenberg AJ, Hudis CA (2016). Obesity and cancer mechanisms: tumor microenvironment and inflammation. J Clin Oncol.

[13] Voegeli D (2020). Intertrigo: causes, prevention and management. Br J Nurs.

[14] Ault KA (2006). Epidemiology and natural history of human papillomavirus infections in the female genital tract. Infect Dis Obstet Gynecol.

